# Gastrocolic fistula after laparoscopic sleeve gastrectomy: Case report and literature review

**DOI:** 10.1016/j.ijscr.2019.11.043

**Published:** 2019-11-27

**Authors:** M. Khalid Mirza Gari, Mohammed S. Foula, Ahmed Eldamati, Saeed Alshomimi, Hazem Zakaria

**Affiliations:** Department of Surgery, King Fahad University Hopsital, Imam Abdulrahman Bin Faisal University, Dammam, Saudi Arabia

**Keywords:** Bariatric surgery, Sleeve gastrectomy, Gastrocolic fistula, Leakage, Complications

## Abstract

**Introduction:**

Laparoscopic sleeve gastrectomy is a popular bariatric procedure. Leakage after sleeve gastrectomy is the cornerstone for most of its related morbidity and mortality. Gastrocolic fistula is a rare complication resulting from chronic leak after laparoscopic sleeve gastrectomy.

**Case presentation:**

We report a case of 32-year-old male who underwent laparoscopic re-sleeve gastrectomy for weight regain after initial uneventful laparoscopic sleeve gastrectomy 3 years back. He presented to emergency department by septic shock secondary to leakage after sleeve gastrectomy. CT abdomen with IV contrast and oral gastrograffin confirmed post sleeve gastrectomy leak. Emergency diagnostic laparoscopy revealed a huge abscess cavity containing pus and dark fecal material and altered blood. A long leak was identified with eversion of gastric mucosa. Tubular structure connecting the upper part of the stomach and the colon was found which turned out to be a gastrocolic fistula. It was controlled by endoscopic linear stapler. After 6 weeks, a definitive open esophago-jeujonostomy with total gastrectomy was done successfully after difficult attempt of laparoscopic intervention. The patient was discharged home in a stable condition.

**Conclusion:**

A high index of suspicion is important in detection of rare complications after laparoscopic sleeve gastrectomy including gastrocolic fistula. Complete laparoscopic resection of gastrocolic fistula is preferred. Gastrectomy might be the definitive surgery.

## Highlights

• Leakage after sleeve gastrectomy is the cornerstone for most of its related morbidity and mortality.
• Gastrocolic fistula is a rare complication resulting from chronic leak after laparoscopic sleeve gastrectomy.
• A high index of suspicion is important in detection of rare complications including gastrocolic fistula.
• Complete laparoscopic resection of gastrocolic fistula is preferred.
• Gastrectomy might be the definitive surgery.
• Re-do bariatric surgery should be only done by an expert bariatric surgeon with multidisciplinary team in a specialized center.

## 1 Introduction

Laparoscopic sleeve gastrectomy (LSG) is a popular bariatric procedure [1]. Despite the innocent approach of the procedure, it can be accompanied by multiple serious complications. Postoperative leakage is responsible for most of LSG related morbidity and mortality [2].

Patients with postoperative leakage present differently. Usually, they present very early after procedure. In others, postoperative leakage may occur several weeks postoperative. High index of suspicion is crucial for diagnosis.

We report a case of gastrocolic fistula after laparoscopic re-sleeve gastrectomy. This work is reported in line with SCARE criteria [3].

## 2 Case presentation

A 32 year-old male, who underwent laparoscopic re-sleeve gastrectomy for morbid obesity six weeks prior to current presentation, presented to emergency department (ED) complaining of two-day history of recurrent moderate amount of coffee ground vomiting, multiple attacks of melena, inability to tolerate food, generalized abdominal pain mainly epigastric and generalized body fatigue.

He did not have any comorbidities. Three years back, he underwent an uneventful LSG as his body mass index BMI was 42 kg/m^2^ (136 kg, 180 cm). He lost 32 kg (58% of excess body weight, BMI 32 kg/m^2^) over two years. Unfortunately, he regained 15 kg afterwards, and his BMI reached 36.7 kg/m^2^. Therefore, he underwent laparoscopic re-sleeve gastrectomy in another hospital six weeks prior to current presentation. He was discharged after two days.

**Fig. 1:**
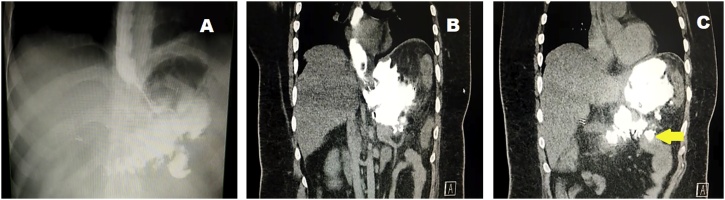
A: Preoperative water-soluble study showing leaking of contrast outside the stomach. B, C: Preoperative CT abdomen with IV and oral contrasts confirming presence of leakage with complete collapse of the stomach. The yellow arrow showing an area of suspicion.

Upon presentation, the patient looked ill, toxic and disoriented. He was tachycardic (110 beats/minute), hypotensive (90/55 mmHg), and feverish (39.5 °C). Abdominal examination revealed rigidity all over the abdomen and epigastric tenderness with no bowel sounds. Laboratory investigations showed leukocytosis with neutrophilia. Computed tomography (CT) of abdomen with intravenous and oral contrasts confirmed leakage (Fig. 1). He was diagnosed as septic shock secondary to leakage after sleeve gastrectomy with Wernicke’s encephalopathy.

**Fig. 2:**
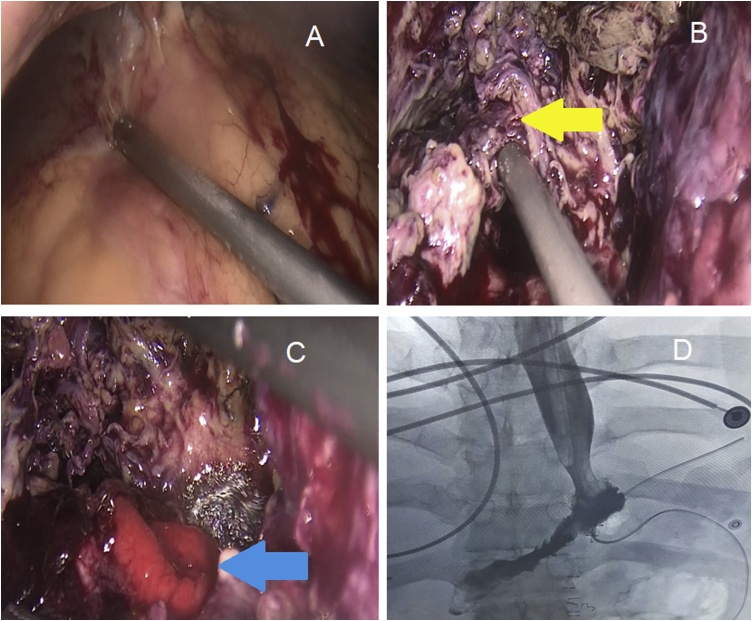
A: Huge abscess formed by liver, transverse colon and omentum. B: The yellow arrow indicating a tubular structure connecting the sleeved stomach (right) with the transverse colon (left); the gastrocolic fistula. C: The blue arrow indicating the site of leak with eversion of gastric mucosa. D: Water-soluble study after esophago-jejunostomy; the definitive procedure showed no residual leakage.

Image-guided percutaneous drainage failed due to close proximity of the transverse colon to the abscess cavity. He was shifted after resuscitation to the operating theater. After general anesthesia and endotracheal intubation, the patient was in supine position at 30° anti-Trendelenburg with abducted legs. Examination under anesthesia revealed epigastric fullness. Pneumoperitoneum was done using Veress needle in Palmer’s point. First trocar was introduced under vision 20 cm below xiphisternum, two centimeters to the left of midline. The second and third trocars were inserted at midclavicular line 15 cm below costal margin left and right respectively. Emergency diagnostic laparoscopy showed no free fluid collection in the abdomen. The greater omentum and transverse colon were walling off a huge abscess with failure of identification of the sleeved stomach. Meticulous blunt dissection was done to reach the gastro-esophageal junction (GEJ) revealing large amount of pus, dark fecal material, and altered blood from the abscess cavity. A large area of leakage was identified with eversion of the gastric mucosa just distal to GEJ. Dissection of the distal part was difficult due to severe adhesions. A tubular structure connecting the stomach and the transverse colon was dissected carefully, which turned out to be a gastrocolic fistula (Fig. 2). It was excised using laparoscopic linear stapler. Three drains were inserted; around the area of the disrupted suture line, in the sub-phrenic space and in the pelvic cavity. Feeding jejunostomy was created laparoscopically.

Postoperatively, he was kept under total parenteral nutrition followed by jejunostomy feeding. Empirical antibiotics and antifungals were changed according to fluid culture results. Other medications included proton pump inhibitor, analgesia and anticoagulants.

After 6 weeks, a definitive open esophago-jejunostomy with total gastrectomy was performed successfully after failed attempt of laparoscopic intervention. Water-soluble contrast study confirmed the continuity of the bowel with no residual leak. The patient was discharged home in a stable condition. He is now following regularly in the outpatient clinic.

## 3 Discussion

The incidence of obesity is increasing in the Saudi society leading to increased demand on bariatric procedures. Due to its simplicity and efficacy, LSG gained more popularity and became one of the most commonly performed bariatric procedures worldwide [2].

Postoperative leakage is the cornerstone complication after LSG. The incidence of postoperative leakage ranges from 1 to 7% [4]. It increases after revisional LSG [1]. It is categorized into acute (within one week), early (1–6 weeks), late (6–12 weeks) and chronic (more than 12 weeks) [2].

Gastrocolic fistula is an uncommon surgical entity. It usually occurs due to malignant tumors of stomach or colon, peptic ulcer disease, trauma or severe intra-abdominal infection [[5], [6], [7]]. Gastrocolic fistula is a rare reported complication after sleeve gastrectomy resulting from persistent intra-abdominal infection caused by postoperative leakage [8]. Its management consists mainly of resuscitation, drainage of any co-existing intra-abdominal abscess and treatment of malnutrition status followed by definitive procedure. Intra-abdominal abscess drainage can be image guided, laparoscopic, open or rarely trans-gastric. Endo-luminal management including stent and over the scope clip may help. The definitive surgical management entails excision of fistula. Gastrectomy and/or colectomy is rarely required.

To the best of our knowledge, only four similar cases were reported. Trelles et al. reported gastrocolic fistula after re-sleeve gastrectomy. Endoscopic stent failed and the fistula was resected laparoscopically [5]. Bhasker et al. reported a gastrocolic fistula after primary LSG that was managed by laparoscopic excision [9]. Garofalo et al. tried the endoscopic over-the scope clips but failed and then laparoscopic resection of the fistula was performed successfully [1]. Nguyen et al. performed total gastrectomy, esophago-jejunostomy and subtotal colectomy to manage gastrocolic fistula after primary LSG [8].

## 4 Conclusion

A high index of suspicion is important in detection of rare complications after laparoscopic sleeve gastrectomy including gastrocolic fistula. Complete laparoscopic resection of gastrocolic fistula is preferred. Gastrectomy might be the definitive surgery. Re-do bariatric surgery should be done by an expert bariatric surgeon with multidisciplinary team in a specialized center.

## Funding

No funds or sponsors.

## Ethical approval

Case reports are exempted from ethical approval according to policies of Imam Abdulrahman Bin Faisal University.

## Consent

Witten informed consent was obtained from the patient for publication of this case report.

## Author contribution

Dr. M Khalid Mirza Gari: main author, reviewing article.

Dr. Mohammed S. Foula: writing the paper, corresponding author.

Dr. Ahmed Eldamati: writing the paper.

Dr. Saeed Alshomimi: reviewing article.

Dr. Hazem Zakaria: reviewing article.

## Registration of research studies

None.

## Guarantor

Mohammed S. Foula.

## Provenance and peer review

Not commissioned, externally peer-reviewed.

## Declaration of Competing Interest

No conflicts of interest.
